# Analysis of Transcription Factor mRNAs in Identified Oxytocin and Vasopressin Magnocellular Neurons Isolated by Laser Capture Microdissection

**DOI:** 10.1371/journal.pone.0069407

**Published:** 2013-07-24

**Authors:** Madison Humerick, Jeffrey Hanson, Jaime Rodriguez-Canales, Daniel Lubelski, Omar M. Rashid, Yasmmyn D. Salinas, YiJun Shi, Todd Ponzio, Raymond Fields, Michael R. Emmert-Buck, Harold Gainer

**Affiliations:** 1 Laboratory of Neurochemistry, National Institute of Neurological Disease and Stroke, National Institutes of Health, Bethesda, Maryland, United States of America; 2 Laser Microdissection Core, National Cancer Institute, National Institutes of Health, Bethesda, Maryland, United States of America; Pisces Therapeutics, United States of America

## Abstract

The oxytocin (Oxt) and vasopressin (Avp) magnocellular neurons (MCNs) in the hypothalamus are the only neuronal phenotypes that are present in the supraoptic nucleus (SON), and are characterized by their robust and selective expression of either the Oxt or Avp genes. In this paper, we take advantage of the differential expression of these neuropeptide genes to identify and isolate these two individual phenotypes from the rat SON by laser capture microdissection (LCM), and to analyze the differential expression of several of their transcription factor mRNAs by qRT-PCR. We identify these neuronal phenotypes by stereotaxically injecting recombinant Adeno-Associated Viral (rAAV) vectors which contain cell-type specific Oxt or Avp promoters that drive expression of EGFP selectively in either the Oxt or Avp MCNs into the SON. The fluorescent MCNs are then dissected by LCM using a novel Cap Road Map protocol described in this paper, and the purified MCNs are extracted for their RNAs. qRT-PCR of these RNAs show that some transcription factors (RORA and c-jun) are differentially expressed in the Oxt and Avp MCNs.

## Introduction

The central nervous system is composed of many cellular phenotypes which play diverse and distinct roles in brain functions [1], [2], [3]. In order to understand the mechanisms that lead to the development and maintenance of these specific cellular phenotypes it is first necessary to have methods that allow for the enrichment and purification of specific cell phenotypes from the heterogeneous populations of cell-types that exist in the brain. This critical step is a prerequisite for the analysis of the unique molecular properties of specific neurons of interest. In this regard, the magnocellular neurons (MCNs) located in the supraoptic nucleus (SON) in the mammalian hypothalamus offer a unique experimental system to work out strategies to approach the problem of isolating identified neuronal populations for subsequent molecular analysis. The mammalian SON is particularly useful for such studies since it contains principally two neuronal phenotypes, the oxytocin (Oxt) and vasopressin (Avp) synthesizing MCNs which are found in approximately equal numbers in the SON [2], [4], [5].

There are many physiological differences that exist between the Oxt- and Avp-MCNs in the hypothalamus [2], [4], [6], but the most prominent distinguishing feature between these phenotypes is their robust and selective expression of the two neuropeptide genes [2], [7]. Historically the neurohypophysial peptide hormones, Oxt and Avp, are best known for their roles in the periphery to stimulate milk let-down during lactation and induce uterine contractions during parturition, and to regulate systemic salt and water balance, respectively [2], [7], [8]. However, more recently, these neuropeptides have also been shown to also have important physiological functions and behavioral effects in the central nervous system [2], [9], [10], [11], [12], [13].

The MCNs in the adult SON are relatively large, about 20–25 µm in diameter [4]. Previously, we were able to isolate the individual MCNs from the SON by gentle enzymatic dissociation followed by manual suction into micropipettes, and their phenotypes were identified by single cell RT-PCR to assess their Oxt or Avp mRNA contents [14], [15]. From these studies a third MCN phenotype was identified. While 97% of the MCNs in the SON expressed either Oxt or Avp mRNAs in a highly selective manner as expected for the two major phenotypes, about 3% of the MCNs in the normal SON were shown to coexpress both mRNAs in approximately equal amounts. Previously we identified the phenotypes by single-cell RT-PCR and could pool the remaining RNAs from either the Oxt or Avp MCNs to produce Oxt- and Avp –MCN specific cDNA libraries [16]. The above strategy was useful for single-cell RT-PCR studies, but does not produce the total amount of high quality, cell-type specific RNA that is necessary for quantitative RT PCR studies.

Recently, we produced recombinant Adeno-Associated Viral (rAAV) vectors that contain Oxt and Avp promoters attached to EGFP reporters that are selectively expressed in either Oxt or Avp MCNs, respectively [17], [18]. By stereotaxic injection of these rAAVs into the rat SON in vivo, it has been possible to unequivocally identify the fluorescent Oxt and Avp MCNs in frozen sections of the SON. In this paper, we make use of these rAAVs to identify the individual MCN phenotypes in order to isolate these two phenotypes from the rat SON by laser capture microdissection (LCM), and to analyze the differential expression of several of their specific mRNAs by qRT-PCR.

## Materials and Methods

### Animals

Adult male Sprague-Dawley rats (270 g–370 g) obtained from Charles River Laboratories (Wilmington, MA) were maintained under normal laboratory conditions (temperature: 21–23°C, 12 h light-dark cycles with light on at 6∶00 AM) with access to unlimited food and drinking water. Following surgical procedures, rats were caged individually.

### Ethics Statement

All procedures were carried out in accordance with the National Institutes of Health (NIH) guidelines on the care and use of animals and an animal study protocol No. 1278 approved by the National Institute of Neurological Disorders and Stroke (NINDS) Animal Care and Use Committee.

### Recombinant Adeno-associated Viral (rAAV) Constructs that Drive Cell-type Specific Expression in Oxt or Avp MCNs

**Figure 1:**
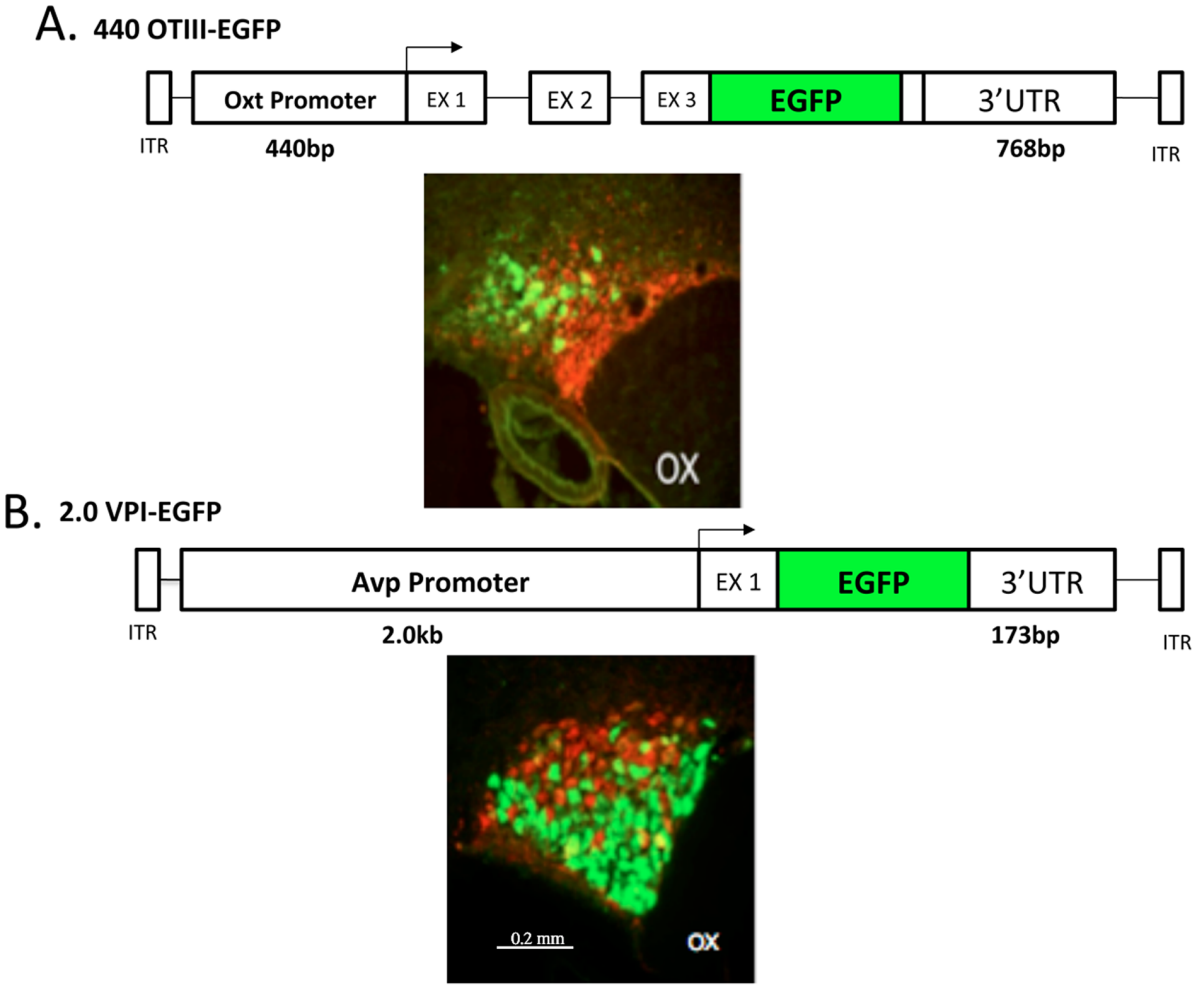
Specificity of Expression of the Oxytocin and vasopressin rAAV constructs in the SON. A. The 440OTIII-EGFP rAAV that was injected into the SON contains 440 bp of the promoter sequence upstream of the transcription start site (TSS) of the oxytocin gene, all three exons, fused to the EGFP sequence within the 3rd exon (EX 3), followed by 768 bp of the 3′UTR of the oxytocin gene [17]. The immunohistochemical (IHC) image shown below the construct shows that the EGFP from the 440 OTIII-EGFP virus is specifically expressed in Oxt MCNs in the dorsal SON. The green color represents the fluorescence of the EGFP immunoreactivity in Oxt MCNs and the red fluorescence depicts the PS 41 antibody immunoreactivity in the Avp MCNs. Scale line for both panels A and B is shown in lower panel B. B. The 2.0VPI-EGFP rAAV that was injected contains 2.0 bp of the promoter sequence found upstream of the vasopressin gene TSS, exon 1 of the vasopressin gene, fused to the EGFP sequence, and 173 bp of the 3′UTR downstream of the Avp gene [18]. The immunohistochemical (IHC) image shown below the construct shows that the EGFP from the 2.0VPI-EGFP virus is specifically expressed in Avp MCNs in the ventral SON. The green color represents the fluorescence of the EGFP immunoreactivity in Avp MCNs and the red fluorescence depicts the PS 38 antibody immunoreactivity in the Oxt MCNs. Abbreviation: OX, optic chiasm.

The procedures used to produce rAAVs that drive cell-type specific expression of EGFP in Oxt or Avp MCNs have recently been described in detail in Fields et al. [17] for Oxt and in Ponzio et.al. [18] for Avp. In the present paper, we use two rAAV constructs to identify the Oxt and Avp MCNs in the SON. These were the 440OTIII-EGFP rAAV which is selectively expressed in Oxt MCNs [17], and the 2.0VPI-EGFP rAAV which is selectively expressed in Avp MCNs [18]. Fig. 1 illustrates the structures of these two rAAV constructs that were sterotaxically injected into the SON, and below each structure an example of their cell-type specific expression of EGFP (green fluorescence) in the Oxt MCNs (Fig. 1A) or in the Avp MCNs (Fig. 1B) is shown. The injected 440OTIII-EGFP rAAV had a titer of 3×10^13^vg/ml, and the injected 2.0VPI-EGFP rAAV had a titer of 1.3×10^13^vg/ml.

### Raav Injection Methods

Details about the stererotaxic injection techniques have been described elsewhere [17], [18]. Briefly, prior to surgery the rats were anesthetized with 5% isoflurane (Baxter) in air using a Stoelting gas anesthesia adaptor for stereotaxic instruments (David Kopf Instruments, Tujuna, CA, USA). The rats were placed in the stereotaxic instrument and the hair on top of the scalp was shaved. The scalp was sterilized by two rounds of application of betadine, followed by a 50∶50 betadine/ethanol mixture, followed by 70% ethanol. A 15–20 mm incision was made using a size 15 scalpel starting rostral to bregma and ending just caudal to lambda. Ensuring a flat skull position was done by determination that both bregma and lamda were on the same horizontal plane. Holes were drilled into the skull in order to lower intracerebral 30-guage syringe needles over the SON bilaterally [19]. The stereotaxic coordinates were determined using the Paxinos and Watson atlas [20] and were: 1.3 mm posterior to bregma, 1.8 mm lateral on each side, and 8.8 mm (for rats between 270–320 grms) or 8.9 mm (for rats between 321–370 grms) dorsal to ventral. The oxytocin (440OTIII-EGFP) and vasopressin (2.0VPI-EGFP) specific rAAV viruses were injected bilaterally over the SON in vivo by convection enhanced delivery [21] at a rate of 0.30 µl/min for ten minutes. Following these procedures, the incision was closed using interrupted sutures (Ethilon, Nylon Suture, Black Monofilament 4-0) and Ketoprofen was administered subdermally (5 mg/kg, diluted in 0.9% NaCl). Rats were then kept individually in cages (18′′/9′′/9′′–l/w/h) for the subsequent two weeks.

### Tissue Preparation for Laser Capture Microdissection (LCM)

**Figure 2:**
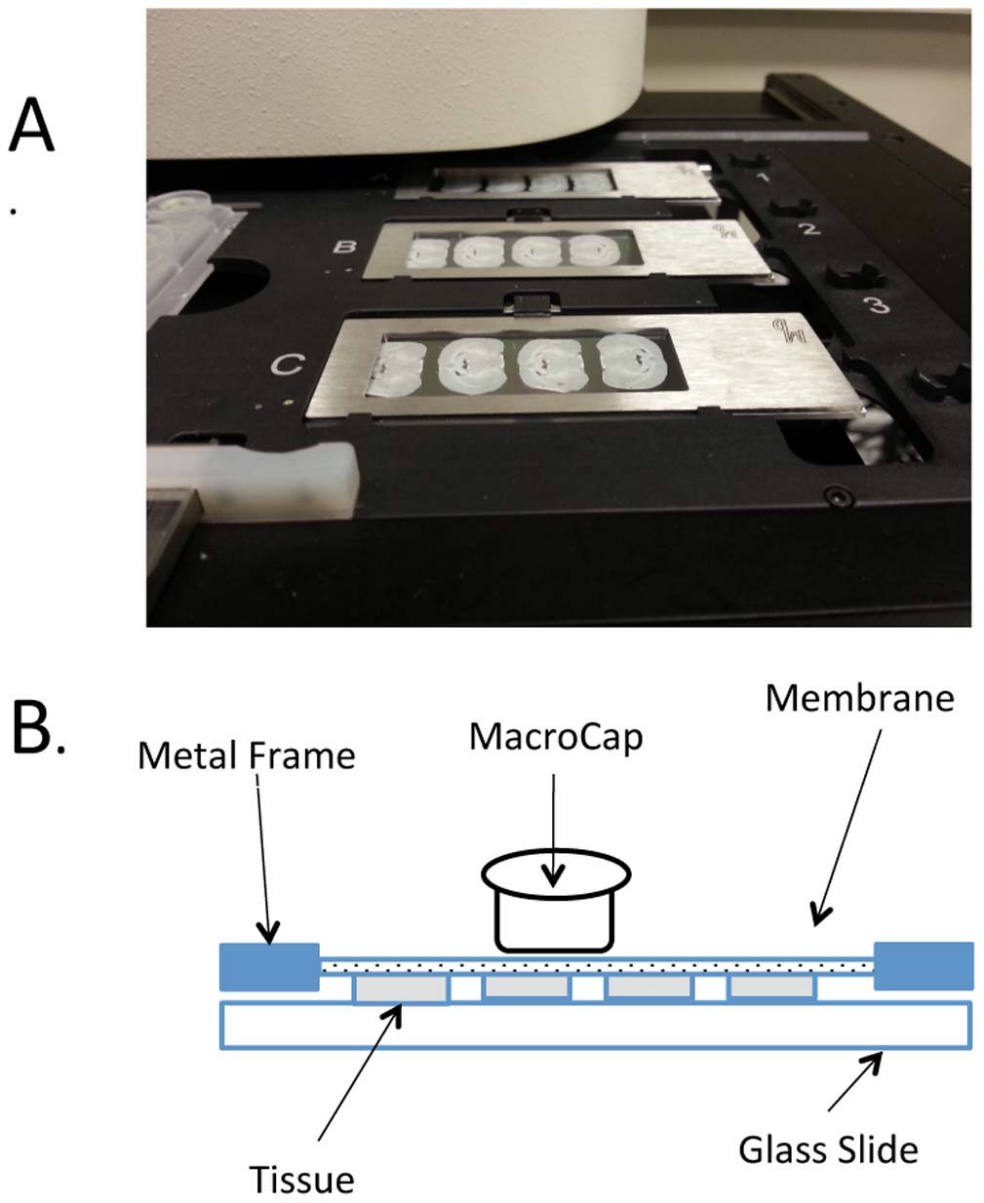
View of brain sections on the Frame metal slide LCM slides, and the LCM Cap position. A. Illustration of three metal frame membrane slides each containing four hypothalamic sections mounted on the stage of the Arcturus XT instrument ready for LCM. B. Schematic view of the Metal Frame Membrane slide and LCM cap. With these slides the LCM cap sits directly on the membrane without touching the tissue. This allows the cap to sit evenly without being affected by any folds in the tissue and adjustments to the laser’s power and alignment usually do not need to be made. Use of the Macro LCM caps also reduces the danger of contaminating the sample with surrounding tissue.

Two weeks following the rAAV injections, the rats were anesthetized using isoflurane and sacrificed by decapitation. Their brains were quickly removed from the skull, and the cerebellum and brain stem were removed and discarded. The brain block containing the hypothalamus was then rapidly frozen on dry ice and stored at −80°C until sectioning was done. For details about this procedure see Table S1. Briefly, the frozen brain was mounted onto Tisue-Tek® OCT compound (Sakura Finetek USA, Torrance, CA) on a chuck with the caudal portion of the brain placed down on the chuck, and the brain on the chuck was placed in the cryostat (Reichert-Jung 2800, Frigocut, Heidelberg, Germany) for twenty minutes to equilibrate the brain with a chamber temperature (CT) of −20°C and an object temperature (OT) of −16°C. Four, 12 µm thick sections of the SON region were cut and placed on polyethylene naphthalate (PEN) Membrane Frame slides (Life Technologies, Grand Island, NY, Cat. # LCM0521) under RNAase-free conditions. Four brain sections were usually mounted on a single PEN membrane slide (Fig. 2A), and between 25 and 30 slides were made from a single brain specimen for up to 120 total sections. Slides were stored immediately in a slide box embedded in dry ice, and the slide box was placed in the −80°C freezer until the slides were used for LCM (slides were used within one month of being sectioned).

### Laser Capture Microdissection (LCM)

Slides were kept in their slide box on dry ice until the dehydration step for visualization on the LCM instrument. Before LCM, the sections were fixed and dehydrated with ethanol and xylene under RNase free conditions, as described previously with some modifications (Luo et al., 1999); the slides were thawed for 30 seconds in 75% ethanol, and further dehydrated by sequential immersion in 95% ethanol for 5 sec, twice in 100% ethanol for 5 sec, followed by xylene for 30 sec. The slides were left in a hood to allow the xylene to evaporate for 3 minutes. All solutions were made with DEPC water. Three slides can be placed in the Arcturus XT LCM instrument at one time, and therefore, only three slides were dehydrated for microdissection at one time. The rest of the slides were stored on dry ice until they were needed. The ethanol fixation technique was optimized for the best visualization of the fluorescent cells under the LCM microscope and the highest RNA yield and quality (see Results and Table 1).

**Table 1:** RNA Yield and Quality.

*Tissue Fixation*	*Yield*	*Quality (RIN #)*
Fresh Frozen Tissue	764.7 ng	7.3
Ethanol and Xylene	356.1 ng	8.3
4% PFA, Ethanol, and Xylene	170.7 ng	1.0

**Figure 3:**
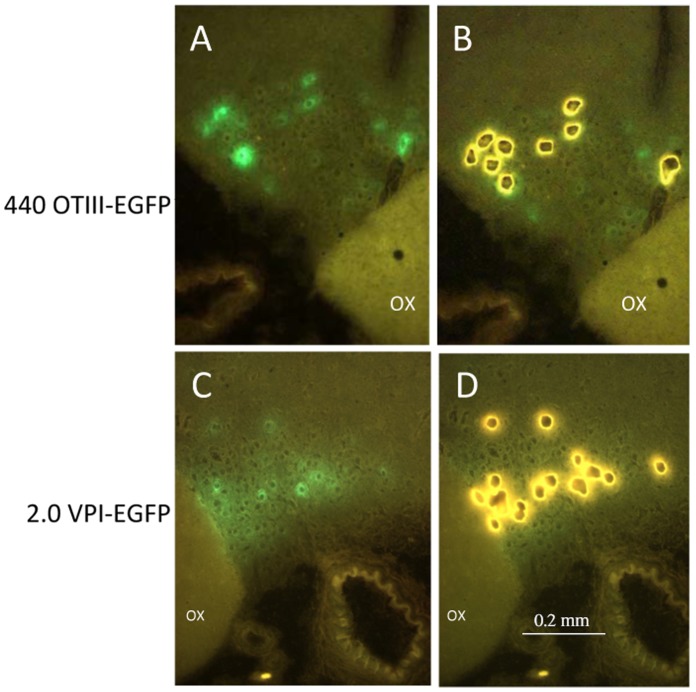
LCM sample dissection as viewed through the Arcturus XT fluorescent LCM microscope. A. View of 440OTIII-EGFP fluorescent expression in oxytocin MCNs in the SON on PEN Membrane Metal Frame slides. 15× magnification. Abbreviation: OX, optic chiasm. B. View of fluorescent Oxt MCN ghosts that have been captured and cut out by LCM. 15× magnification. C. View of 2.0VPI-EGFP fluorescent expression in Avp MCNs in the SON on PEN Membrane Metal Frame slides. 15× magnification. D. View of fluorescent Avp MCN ghosts that have been captured and cut out by LCM. OX represents the optic chiasm. 15× magnification. Scale line shown in panel D is representative for all panels.

LCM was performed using the above-mentioned Arcturus XT (Life Technologies, Washington DC 20001) laser-microdissection system, with fluorescence, immediately after fixation and dehydration of the slides. The EGFP-fluorescent neurons were microdissected using CapSure Macro LCM Caps (Life Technologies, Cat. # LCM0211). The PEN membrane frame slide was loaded on the instrument with the tissue side down and a clean glass slide underneath it acting as support. Figure 2B illustrates how the LCM cap sits on the opposite side of the membrane than the tissue sections, allowing for close contact between the cap surface and the PEN membrane of the slide and reducing chance of nonspecific pickup. Figure 3 shows the fluorescence of the MCNs transduced by the AAVs as visualized on the Arcturus XT Fluorescent LCM microscope before (Figs. 3A and C) and the excised cells after (Figs. 3B and D) the laser microdissection. The rat SON is completely visualized in the 20× objective view of the Arcturus XT LCM microscope and this allowed for the development of the LCM Cap Road Map method for the systematic microdissection of multiple SON neurons from many serial sections from the same brain specimen onto the same cap, thereby increasing the total number of specific target cells to be obtained per SON sample (see Fig. S1 and Table S2). The method consisted of subdividing the LCM Macro Cap into a maximum of 51 subregions and then collecting the fluorescent Oxt or Avp MCNs from each section by placing the cap sequentially in each cap subregion (Fig. S1A and Table S2). The cap subregion sequence was designed using a cap road map for the LCM operator; keystroke commands in the Arcturus XT software were used to manually move the cap to the specific region for the collection of the neurons from each brain section (See keystrokes in Fig. S1A and detailed description in Table S2). Figure S1B illustrates a completed Macro LCM cap containing isolated MCNs from an SON after using the LCM Cap Road Map method.

### RNA Isolation and Quality Assessment

The RNA was isolated using the Picopure RNA Isolation Kit (Life Technologies, Cat. #KIT0204). The Macro cap with the microdissected fluorescent cells (see Fig. S1B) was immediately seated into a 0.5 mL tube containing 50 µL of PicoPure Extraction buffer and incubated for 30 minutes. When multiple caps for an individual rat were collected, the buffer from each cap containing the lysed cells was pooled in a 1.5 mL microcentrifuge tube before continuing with the isolation. After isolation, the RNA’s concentration was measured using a Nanodrop spectrophotometer (ND-1000, Thermo Scientific, Wilmington, DE). The RNA samples were analyzed using a 2100 Bioanalyzer (Agilent Technologies, Santa Clara, CA 95051) to obtain RNA integrity numbers (RIN #s) as measures of their quality [23]. These data are shown in Table 1.

### Quantitative RT-PCR

qRT-PCR was performed using the StepOnePlus Real Time PCR System (Applied Biosystems) with the QuantiTect SYBR Green RT-PCR kit (Qiagen, Cat.# 204243). All expression levels of genes of interest were normalized to the housekeeping gene, glyceraldehyde-3-phosphate dehydrogenase (GAPDH). The following genes were analyzed: oxytocin, vasopressin, retinoic acid orphan receptor alpha (RORA), CRE binding protein 3 (CREB3), circadian locomoter output cycles kaput (Clock), and c-jun, and the primers for the PCR reactions are shown in Table S3. Gene mRNA sequences were obtained using the NIH NCBI website (http://www.ncbi.nlm.nih.gov/gene/), and primers were designed using the Invitrogen OligoPerfect Design Program (http://tools.invitrogen.com/content.cfm?pageid=9716). The sequences were checked for specificity using Basic Local Alignment Search Tool (BLAST) on the National Center for Biotechnology Information (NCBI) website (http://blast.ncbi.nlm.nih.gov/Blast.cgi). Primers were validated by qRT-PCR and gel electrophoresis using prepared control rat RNA. The reactions were set up according to the manufacturer’s handbook: Step 1∶50°C for 30 min, Step 2∶95°C for 15 min, Step 3∶40 cycles of 95°C for 15 sec, 60°C for 30 sec, and 72°C for 15 sec. The annealing temperature was set at 60°C for 30 sec for all primers. Eight nanograms of RNA were used in each of the reactions and the results in Ct values are shown in Table S4. The fold differences of each species of RNA found in the Oxt versus Avp MCNs presented in Table 2 were determined by sequential log transformation of the Ct values, mean centering, and autoscaling calculations as described in [25]. The statistically significant differences shown in Table 2 were determined using the Mann-Whitney Test [26].

**Table 2:** Calculated Fold Differences of Oxt vs. Avp MCN mRNA.

mRNA	Observation	Mann-Whitney Test p-value
Oxt	41.7× greater in Oxt MCNs compared to AvpMCNs in the SON	0.046850
Avp	11× greater in Avp MCNs compared to Oxt MCNs inthe SON	0.046850
RORA	3.4× greater in Oxt MCNs compared to AvpMCNs in the SON	0.046850
CREB3	No difference between Oxt vs AvpMCNs in theSON	0.619300
Clock	No difference between Oxt vs AvpMCNs in the SON	0.619300
c-jun	2.1× greater in Oxt MCNs compared to AvpMCNs in the SON	0.046850

## Results

### Identification of the Oxt and Avp MCNs in the Rat SON for Laser Capture Microdissection

In this paper we use rAAVs containing previously characterized cell-type specific promoters in the Oxt and Avp genes [17], [18] to drive the expression of EGFP selectively in the Oxt or Avp MCNs in the rat SON (see Fig. 1). Two weeks after stereotaxic injection of the specific rAAVs into the rat SONs of rats the Avp or Oxt MCNs in the SONs become robustly and selectively fluorescent and are readily visualized using either the fluorescent Arcturus or the Veritas microscope for the LCM of the individual cells.

#### Evaluation of fixation methods for LCM

We evaluated three treatments of the frozen brain sections on the slides for their effects on RNA quality. This included 1) RNA isolated from frozen brain sections that were scraped into the extraction buffer with no fixation at all, 2) ethanol fixation and xylene dehydration only, and 3) 4% paraformaldehyde fixation of fresh frozen slides for 5 minutes with subsequent dehydration for 30 sec in 75% ethanol, and further dehydration by sequential immersion in 95% ethanol for 5 sec, twice in 100% ethanol for 5 sec, and finally xylene for 30 sec (modified from [22]). The paraformaldehyde (PFA) fixation obscured visualization of the cell boundaries and cell nuclei, and hence, was not suitable for cutting and capturing single cells. In addition, the PFA fixation produced one half of the RNA yield of the short ethanol fixation and a much poorer quality of RNA (i.e., with a much lower RNA integrity number (RIN #), see Table 1). The purified RNA samples were analyzed using a 2100 Bioanalyzer (Agilent Technologies, Santa Clara, CA 95051) to obtain RNA integrity numbers (RIN #s) as measures of their quality [23]. These data are shown in Table 1, and from these data we concluded that ethanol dehydration with subsequent xylene dehydration allowed for the best visualization of the cells under fluorescent microscopy as well as higher quality RNA with RINs >6. (see also [24]).

### Laser Capture Microdissection: The Cap Road Map Protocol

The LCM Cap Road Map protocol was used to maneuver the cap used in LCM in an orderly and sequential manner, thereby making the collection process quick and efficient and ensured optimal mRNA extraction and analysis. Other optimization was also done to ensure that the RNA samples had little debris and contamination. Since frame metal slides were used, the tissue sections were mounted facing down and the LCM cap was placed on top of the membrane of the frame metal slide (Fig. 2B). Therefore, there was no direct contact between the cap and the tissue section. This avoided contamination with tissue debris when the cap was repositioned on a new section. By using the Cap Road Map we could dissect and capture 37 regions without overlapping with any previously captured MCNs present on the cap. In order to place the cap on the area to be cut, the cap had to be viewed in the 2× objective to ensure that the area about to be cut would not overlap with tissue that was already on the cap. Using the cap road map, the cap could be moved and 37 regions could be cut and captured without changing the objective, thereby saving a significant amount of time and consequently reducing mRNA degradation. Before using the cap road map, cutting and capturing 37 regions would take about 1.5 hours. Using the Cap Road Map protocol cutting and capturing MCNs from 5–6 slides (which filled 37 regions on the cap) typically took about 45–60 minutes.

A more complete description of the LCM Cap Road Map protocol that we have developed and used here is described in Table S2. Briefly, four brain sections were mounted on a single PEN membrane slide and between 25 and 30 slides were made from a single brain specimen. Three slides at a time are placed in the slide holder of the Arcturus XT (see Fig. 2A), and a single LCM cap is selected to collect the fluorescent MCNs from the sections (Fig. 2B).

The brain areas to be cut and captured (e.g., the SON) were centered and focused on the screen using a 20× objective (see center in Fig. S1A), and their positions were stored according to the LCM Cap Road Map protocol (see Table S2). After returning to the first stored position using the LCM Cap Road Map protocol, the MCNs to be cut and captured were outlined and the cap was placed at the region center (blue box in center of Fig. S1A). The MCNs were then microdissected, by clicking the “cut and capture” button on the Arcturus screen. The first cut and captured MCNs were then at the center of the Macro cap. All subsequent MCNs were microdissected according to the LCM Cap Road Map protocol (see Table S2). Successive MCNs were collected sequentially in a circumferential pattern moving outward from the center subregion, Figure S1B shows a Macro cap in which all 51 regions on the cap were used to capture cells on a Macro cap using the LCM Cap Road Map protocol. Although up to 51 regions can fit on the LCM Macro cap (Fig. S1), because of the lability of the RNA sample, RNA isolation from the MCNs was typically terminated only after 37 subregions were collected on any given cap.

### RNA Quality Analysis

The average number of fluorescent Oxt MCNs per rat that were microdissected from each pair of SONs was 732±51 (sem), and the average number of Avp MCNs per rat isolated from each pair of SONs was 1009±123 (sem) (Table S5). The average amount of RNA per rat SON pair that was extracted from the above dissected MCNs was 103±10 ng and 91±97ng from Oxt and Avp MCNs, respectively. The RIN values for these isolated RNAs prepared from the LCM derived MCNs ranged from 5.5 to 6.8. Compared to the RINs of RNAs obtained from ethanol fixed and dehydrated tissue scrape’s hypothalamic tissue which had a RIN of 8.3 (Table 1), the lower RIN values of the RNA obtained by LCM suggests that there is significant RNA degradation in the dissected single neurons during the LCM procedure. Since RIN values for RNA around 6.0 have been found to be acceptable for use in real-time PCR studies [23] we used only LCM derived RNA samples with RINs equal or greater than 6.0 in our qRT-PCR studies described below and in Tables 2 and S4.

### qRT-PCR Analysis

The four Oxt MCN and three Avp MCN RNA samples shown in Table S4 were selected for qRT-PCR analysis because they met the RIN quality criterion of >6.0. The direct Ct values of these RNA samples obtained by qRT-PCR are shown in Table S4. It is reassuring that the Ct values found for the Oxt mRNA in the LCM-derived Oxt MCN RNAs were substantially lower than the Avp mRNAs (indicating a significant enrichment of Oxt mRNA in these samples) and that the reverse was true for these mRNAs for the LCM-derived Avp MCN samples, as might be expected. The raw Ct data in Table S4 were then analyzed to determine the fold differences in mRNAs between the Oxt and Avp MCNs using the algorithm described in Willems et.al. [25] for all the mRNA species studied. The fold change calculations of these data are shown in Table 2.

Table 2 shows that Oxt mRNA expression was 47.1 times greater in the Oxt MCN –derived RNAs as compared to the Avp MCN-derived RNAs, and Avp mRNA expression was 11 times greater in the Avp MCN-derived RNA compared to the Oxt MCN-derived mRNA. These data demonstrate that the two cell types were significantly enriched by the combined AAV-LCM method used here. Analysis of selected transcription factor mRNAs also showed differential gene expression between the Oxt and Avp MCN-derived RNAs (Table 2). RORA mRNA was 3.4 times greater in Oxt MCNs as compared to Avp MCNs, and c-jun mRNA was 2.1 times greater in the Oxt MCNs compared to the Avp MCNs. In contrast, CREB3 and CLOCK 1 showed no differences in mRNA levels in Oxt versus Avp MCNs.

## Discussion

In this paper, we describe a strategy for the enrichment of specific neuronal phenotypes from the central nervous system (CNS) for the purpose of elucidating their distinct molecular properties. This strategy was developed using the Oxt and Avp MCNs in the SONs in the rat hypothalamus as the targets, but can be applied to any neuronal phenotypes in the CNS. The strategy involves four steps: 1) The sterotaxic injection into specific CNS nuclei (e.g., the SON) of constructs containing cell-type specific gene promoters that drive EGFP reporters which have been packaged into rAAV vectors. 2) The resultant EGFP fluorescence displayed by the specific neurons is then visualized through a fluorescent microscope coupled to a LCM capacity (e.g., the Arcturus XT used here). 3) The fluorescent single cells are dissected by LCM, harvested and pooled by using a novel “LCM Cap Road Map” protocol, and the total RNA of the pooled cells is extracted and purified. 4) Specific mRNAs in the extracted total RNA are then quantitatively evaluated by qRT-PCR.

### Single Cell LCM Technology

LCM technology was first developed at the National Institutes of Health in 1996 for the purpose of selectively obtaining small cancerous tissues surrounded by normal tissues [27] Since that time LCM has been widely used in biomedical research including for the analysis of DNA mutation analysis [28], epigenetics [29], proteomics [30], microRNAs and gene expression [31], [32], [33], [34], [35]. Several studies comparing RNA extracted from whole tissue sections versus specific cells dissected in the tissue by LCM have demonstrated that the specific cells dissected by LCM provide more relevant samples for gene expression analyses [36], [37], [38]. This is particularly true for studies in the central nervous system where there is a great heterogeneity of neuronal phenotypes [3]. Consequently, there have been a number of gene expression and transcriptional profiling experiments in CNS nuclei that have employed LCM to acquire specific brain regions or nuclei for these studies [39], [40], [41], [42].

Several investigators have used LCM methods to obtain single neurons of a given phenotype for gene expression profiling. Many of these have been on postmortem human brain tissues [2], [24], [43], [44], [45] with the recognition that postmortem brain undergoes significant RNA degradation even when quick frozen procedures and ethanol fixations are used [24]. In some studies single cells were isolated by LCM from fresh frozen sections in various brain regions [46], [47], [48] but the quality of the RNAs that were isolated was not rigorously evaluated. In our study, we employed a novel strategy to identify the specific neuronal phenotypes to be dissected by LCM. Our technique allowed for the visualiziation of the endogenous EGFP fluorescence in the Oxt and Avp MCNs in the SON expressed through the transduction of the MCNs by rAAVs containing MCN-specific promoters driving the expression of an EGFP reporter [17], [18]. With this rAAV injection strategy, cryostat sections of fresh frozen tissue with only quick ethanol fixation and dehydration can be used prior to performing the LCM thereby providing better RNA quality than with aldehyde based fixation techniques [32], [49]. Because the virus produces its own mRNA and fluorescent protein, the cells can readily be identified under a fluorescent microscope and the endogenous mRNA is left relatively unperturbed. We chose to use frame metal slides in order to avoid contamination of the cap with debris or tissue surrounding the cut cells. This is because with frame metal slides the tissue samples do not come into direct contact with the cap surface. If there are any folds in the tissue, the cap will still rest flat on the membrane thereby avoiding the need to adjust the laser power to capture the area of interest. Because frame metal slides were used in this study, the cap never came into contact with the tissue and the Macro caps could be used without having the threat of contamination. This allowed more cells to be collected per cap.

### The LCM Cap Road Map Protocol

The LCM Cap Road Map protocol that we developed and describe here allowed for the collection of cells from serial sections on a single LCM cap, thus facilitating the enrichment of the RNA per SON sample. This method is applicable to laser capture microdissection systems using a near infrared laser to transfer the cells from the slide to an LCM cap polymer, such as the current Arcturus XT system used in this study. A major advantage of this method is that objectives do not to have to be changed during the LCM session, thereby requiring less time per LCM session. Since the users know where the tissue samples are on the cap during the entire LCM session, they never need to view the cap at 2× magnification to make sure that the tissue has been placed in the correct area on the cap. This substantially reduces the time to cut and capture the tissue sample at the LCM microscope before placing the sample into RNA extraction (lysis) buffer. Shortening the time for LCM can increase the amount of slides and therefore number of cells that a user can collect without compromising RNA integrity. In addition, the user knows where all the samples have been placed without a fear of overlapping with previously captured tissue. By not overlapping previously cut tissue and using the pattern created by the cap method, the tissue is spread evenly over the entire cap and the cap is never packed too full with the sample (see Fig. S1B). This prevents having to adjust the laser power frequently throughout the LCM session, which also contributes to higher quality RNA (See Methods and Table S2).

### Differential Expression of Transcription Factors in Oxt Versus Avp MCNs

Oxt and Avp MCNs express their respective neuropeptide genes in a very robust and highly selective manner [7]. In this study, we determined whether the Oxt and Avp MCNs that we isolated by the above described strategy could be shown to have differential expression of other genes that are much less robustly expressed. Since transcription factor (TF) mRNAs are known to be present in low copy numbers in cells, we chose to study this class of mRNAs by real-time PCR, as a ‘proof of principal.’ The first mRNAs that we evaluated were the Oxt and Avp mRNAs themselves, both as positive controls and as measures of the degree of purification of the Oxt and Avp MCNs.

The data in Table 2 show that the Oxt MCNs isolated from the SON by LCM contained 41.7-fold greater Oxt mRNA than Avp mRNA, indicating a high degree of enrichment of the Oxt MCNs. The Avp MCNs were also significantly enriched by the LCM procedure so that the Avp mRNA in this sample was 11-fold greater than the Oxt mRNA. Thus, it appears that the Oxt MCNs were more effectively enriched than the Avp MCNs, and that both enrichments were considerably less than the 100–500∶1 ratio expectations that we had based on previous experiments in which we used different single cell isolation methods and different mRNA assays [15]. We do not know the reason for this difference in ratio, but the LCM method could not distinguish the 3% of the MCNs that contain equal amounts of Oxt and Avp mRNAs and therefore these were included in the LCM samples. In contrast, the alternative physical method of cell isolation from the SON could exclude this phenotype [15]. However, this potential contamination of the LCM sample is too small to account for the ratio differences between the methods. A possible explanation is that there is greater contamination of the samples by the LCM method of isolation of the single cells than by the alternative enzymatic dissociation and single cell capture in a microelectrode method that gave a more purified sample [15]. The advantage of the LCM method is that it is possible to isolate more cells of a given phenotype, albeit a less pure population. Nevertheless, the LCM method described here produced sufficient enrichment of the Avp and Oxt MCN phenotypes to evaluate whether any of the TF candidate mRNAs were differentially expressed in each phenotype.

The specific TF mRNAs that we selected for study were RORA, CREB, CLOCK,and c-jun, all of which have been reported to be present in the SON and have been implicated in either Oxt or Avp gene expression [7], [50]. The data in Table 2 show that there was greater expression of RORA mRNA (3.4×) and c-jun mRNA (2.1×) in the Oxt MCNs versus the Avp MCNs (p = 0.047), but no differences in expression were found for the CREB3 and CLOCK TF mRNAs. The biological significance of the greater expression of RORA and AP1 mRNAs in Oxt MCNs is unclear at present, but it is interesting that orphan nuclear hormone receptors such as RORA have been hypothesized to play a role in Oxt gene expression [51], [52]. However, while c-jun has been proposed to be a regulator of Avp gene expression [53], [54] but not Oxt gene expression, the greater expression of this TF in Oxt MCNs does not correlate with this association. Since TFs have many targets in a MCN other than the Oxt or Avp genes, and the expression of any given gene is likely to be determined by more than one TF, it is unlikely that the specific TF’s expression in the cell will reflect only the expression of the specific neuropeptide gene in the cell. In any case, the data in Table 2 give credence to the view that the Oxt and Avp MCNs isolated by the AAV-LCM strategy described in this paper will be useful to identify other genes that are differentially expressed in the MCNs, and therefore can provide a more complete cell-type specific expression profile for these phenotypes.

### Conclusions

In this paper, we have described a strategy to selectively dissect and collect specific neuronal phenotypes in the CNS for analysis. By transducing these neurons using AAVs containing cell-type specific promoters driving he expression of GFP, there is a selective expression of fluorescence only in the specific neuronal phenotypes. These fluorescent neurons are then dissected by the use of LCM and the LCM Cap Road Map protocol described in this paper to give more than 10-fold enrichment of the neuronal phenotype of interest. The RNA isolated from the collected neurons can then be evaluated for their various mRNA species and concentrations by using real-time PCR or microarrays to determine the gene expression profiles of the specific neurons. One caveat to consider is that the RIN values of the RNAs derived from single cell LCM are relatively low, i.e., about 6 or less. This value is acceptable for RT-PCR analysis but is below the usual criterion acceptable for RNA-seq, which is >7 for optimum sequencing results. The lower RIN values for RNA from single cells isolated by LCM as opposed to the higher RIN values (>7), when larger areas such as whole SON is dissected and collected by LCM, suggests that there is some damage to the cellular RNA caused by the lasers in the LCM when applied to such small targets as single neurons. Another issue is whether the expression of an exogenous fluorescent gene in a cell, either by germ line transgenesis or by viral transduction, could alter the endogenous gene expression in the cell. There is no definitive answer to this question at present and this issue should be addressed in future experimental studies.

An alternative approach to obtain single neuronal phenotypes from CNS is to use LCM on cells in transgenic animals which exhibit cell-type specific fluorescence or to use Fluorescence activated Cell Sorting (FACS) to separate and collect the specific fluorescent neurons [55], [56], [57]. FACS usually requires the use of brains of very young animals to avoid the myelin and tight extracellular matrix proteins that are present in adult brains that can interfere with the dissociation of healthy neurons from the CNS. For adult rats and mice, we suggest that the LCM approach described in this paper is the method of choice.

## Supporting Information

Figure S1 **LCM Cap Road Map showing the necessary keystrokes used to maximize use of the space on the cap in the microdissection of multiple neurons from many serial sections of the same brain specimen.** A. The LCM Cap Road Map is shown on the left, indicating the keystrokes necessary to place the centered and focused area to be cut and captured at any of the areas labeled 1–37. The circle superimposed on the road map indicates where the subregions would fall on the actual cap. The blue square is the center of the cap. The green spaces are subregions that will always fit on the cap. The gray spaces may or may not fit on the cap depending on how large the area to be cut and captured is. This map can be used at any objective; the objective used in this study was 20×. Larger objectives will not be able to fit as many subregions on the cap. Once the area to be cut and captured is centered and focused on the screen, left click on the screen, hold down the control key on the keyboard and use the arrow keys to move the screen to the area chosen (1–37). Then, click on the center at region center button on the LCM toolbar. The cap will be moved and placed back on the slide. Click on the cut and capture button on the toolbar, and the area will now be in the location you chose on the map. (For a more detailed description of the LCM Cap Road Map protocol, see Table S2). B. Illustration of an example of a completed Macro cap using the LCM Cap Road Map protocol. If a smaller objective is used, more areas can be cut and captured than are shown in the figure (see text).
(TIFF)

Table S1 **Preparing Fresh-Frozen Tissue Sections for Laser Capture Microdissection.**
(DOC)

Table S2 **Laser Capture Microdissection (LCM) Protocol and LCM Cap Road Map Method.**
(DOC)

Table S3 **Primers used for qRT-PCR of Oxt and AvP MCN mRNA.**
(DOC)

Table S4 **Raw Ct Values for qRT-PCR of Oxt and Avp MCN mRNA.**
(DOC)

Table S5 **Number of Cells Collected by LCM and Amount of RNA Extracted from Each Rat.**
(DOC)
