# Supplementary figures and images for: Analysis of Transcription Factor mRNAs in Identified Oxytocin and Vasopressin Magnocellular Neurons Isolated by Laser Capture Microdissection
Source: PLoS One. 2013 Jul 24;8(7):e69407. doi: 10.1371/journal.pone.0069407 (PMC3722287; doi:10.1371/journal.pone.0069407)

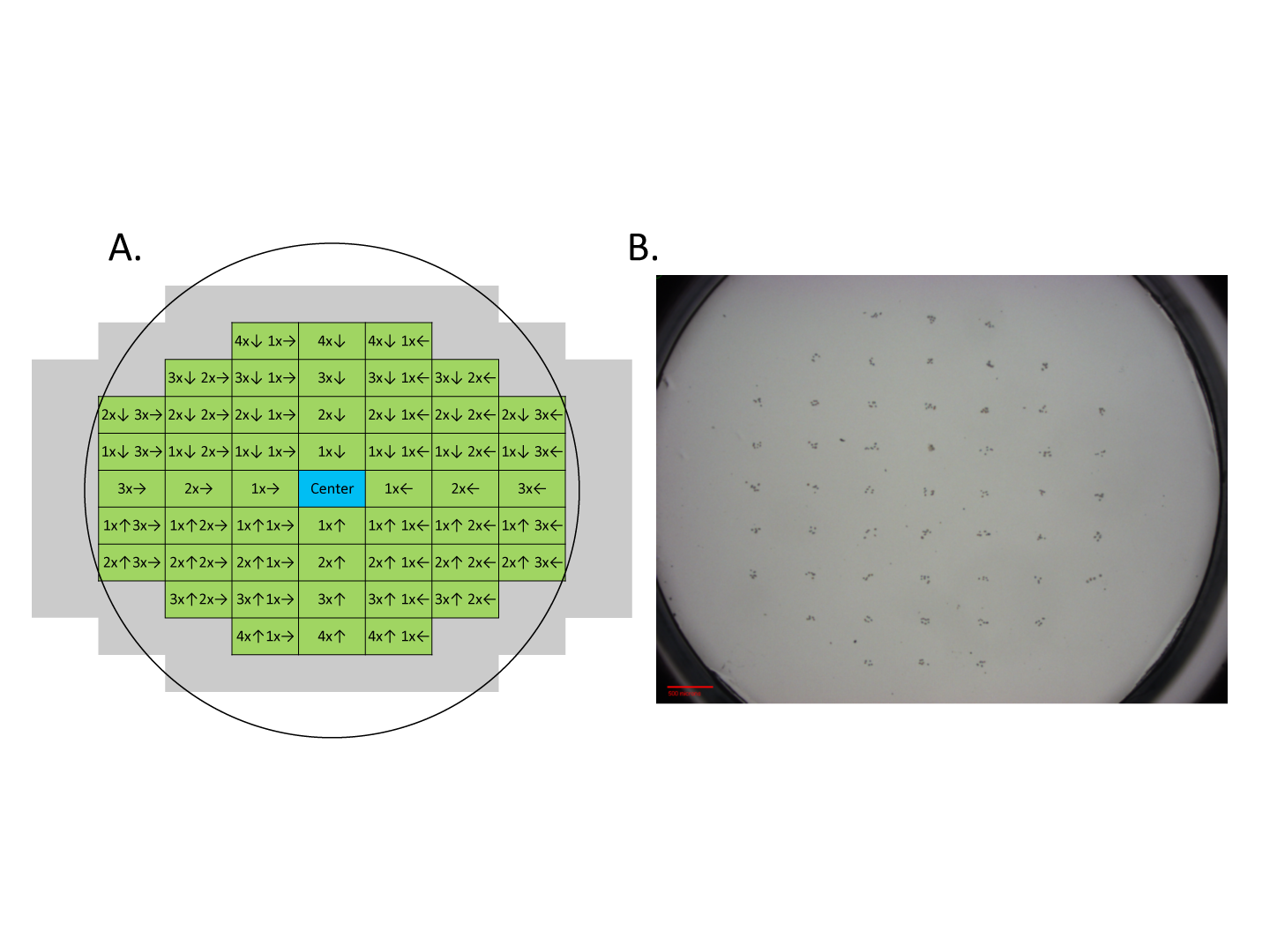

Supplement: Figure S1 — LCM Cap Road Map showing the necessary keystrokes used to maximize use of the space on the cap in the microdissection of multiple neurons from many serial sections of the same brain specimen. A. The LCM Cap Road Map is shown on the left, indicating the keystrokes necessary to place the centered and focused area to be cut and captured at any of the areas labeled 1–37. The circle superimposed on the road map indicates where the subregions would fall on the actual cap. The blue square is the center of the cap. The green spaces are subregions that will always fit on the cap. The gray spaces may or may not fit on the cap depending on how large the area to be cut and captured is. This map can be used at any objective; the objective used in this study was 20×. Larger objectives will not be able to fit as many subregions on the cap. Once the area to be cut and captured is centered and focused on the screen, left click on the screen, hold down the control key on the keyboard and use the arrow keys to move the screen to the area chosen (1–37). Then, click on the center at region center button on the LCM toolbar. The cap will be moved and placed back on the slide. Click on the cut and capture button on the toolbar, and the area will now be in the location you chose on the map. (For a more detailed description of the LCM Cap Road Map protocol, see Table S2). B. Illustration of an example of a completed Macro cap using the LCM Cap Road Map protocol. If a smaller objective is used, more areas can be cut and captured than are shown in the figure (see text). (TIFF) [file pone.0069407.s001.tiff]
