# Supplementary material for: Analysis of Transcription Factor mRNAs in Identified Oxytocin and Vasopressin Magnocellular Neurons Isolated by Laser Capture Microdissection
Source: PLoS One. 2013 Jul 24;8(7):e69407. doi: 10.1371/journal.pone.0069407 (PMC3722287; doi:10.1371/journal.pone.0069407)
Supplement: Table S1 — Preparing Fresh-Frozen Tissue Sections for Laser Capture Microdissection. (DOC) [file pone.0069407.s002.doc]

**Table S1**

**Preparing Fresh-Frozen Tissue Sections for Laser Capture Microdissection**

******Always use RNase-Free technique for tissue to be used for RNA extraction******

--Wear disposable gloves and change them frequently

--Use RNase-free solutions, glassware, and plastic ware

--Use RNase Away on all surfaces and equipment to be used

--Designate a separate area on workbench for RNA work

**Tissue Sample Preparation**

1. Prepare a bucket of crushed dry ice to place fresh brain
2. Anesthetize the rat with 1 mL isofluorane and euthanize the rat by decapitation (using the guillotine)
3. Immediately remove the brain from the skull
4. Remove the cerebellum and brain stem by slicing the brain with a razor blade
5. Place a dime sized portion of the Optimum Cutting Temperature (OCT) embedding compound (e.g., Tissue-Tek®) on a quarter to act as a stage for the freshly sliced brain
6. Place the freshly sliced, caudal portion of the brain down on the OCT and immediately freeze brain on dry ice
7. Store the brain at -80°C until ready to section

**Slide Preparation and Sectioning Fresh Frozen Tissue Sample**

1. Clean cryostat with 100% ethanol
2. Pre-cool the cryostat to: -16°C object temperature (OT) and -20°C chamber temperature (CT)
3. Set cutting thickness to 12μm
4. Take the frozen brain out of the -80°C freezer and mount the caudal aspect of the brain onto on a chuck with OCT with the rostral aspect facing up.
5. Place the chuck with the brain in the cryostat so that its temperature can equilibrate for approximately 20min
   1. During the temperature equilibration, embed a clean slide box (that has been pre-chilled in the -80°C freezer for at least an hour) in a bucket of dry ice
   2. Clean the roll bar and a fresh cutting blade with 100% ethanol and return them to their appropriate place inside the cryostat
6. Place the chuck in the holder of the cryostat and position the brain in the correct orientation
7. Trim the brain until the desired location in the brain is reached
8. Cut the 12μm section and press the membrane of the PEN Membrane Frame slide (Life Technologies, Grand Island, NY, Cat. # LCM0521) to the section and apply gentle pressure for 1-2 seconds
9. Place the slide on top of the cryostat (outside at room temperature) until the next section is cut
10. Put four sections on each membrane slide
11. Immediately place the slide into the slide box after the 4^th^ section has dried on the membrane (can take up to 20 seconds for the section to dry on the membrane)
12. Once all sectioning is complete, store the slide box in the -80°C freezer
13. It is best if slides are used within two days of sectioning, preferably, and no later than one month after sectioning
