# Supplementary material for: Analysis of Transcription Factor mRNAs in Identified Oxytocin and Vasopressin Magnocellular Neurons Isolated by Laser Capture Microdissection
Source: PLoS One. 2013 Jul 24;8(7):e69407. doi: 10.1371/journal.pone.0069407 (PMC3722287; doi:10.1371/journal.pone.0069407)
Supplement: Table S2 — Laser Capture Microdissection (LCM) Protocol and LCM Cap Road Map Method. (DOC) [file pone.0069407.s003.doc]

**Table S2**

**Laser Capture Microdissection (LCM) Protocol and LCM Cap Road Map Method.**

Below is the detailed procedure used collecting individual cells from multiple layers of the Supraoptic Nucleus, and was utilized to conduct the experiments described in the main body of the paper. The protocol is written with the RNA as the target biomolecule for analysis. Included in the procedure are the following: preparing the work area, staining and dehydration of the slides, setting up the instrument, using the software, the first steps of RNA isolation. The procedure can be modified to accommodate the collection of other cell types in regions of interest.

**Materials List**

The PEN Membrane Frame Slides with tissue mounted on them as described in Supplemental Protocol One (S1)

Deep insulated box / cooler

Dry ice

RNase Away or equivalent

GeneAmp Thin-walled Reaction Tube, 500 µl PCR tubes, N8010611, Life Technologies

Rack for PCR tubes

Rack for 2.0 ml tubes

LCM caps (Arcturus CapSure Macro or HS LCM Caps, Life Technologies)

Pipets 100 µl and 20 µl

Pipet tips, filter tips, pre sterilized RNase free

Arcturus PicoPure RNA Extraction Buffer, Life Technologies

2 ml capless collection tubes to use as support tubes

Centrifuge 5415 D, Eppendorf or equivalent

Clean glass slides to use as supports

Lens paper

Pap Jars (Evergreen Scientific, Los Angeles, CA) or 50 ml Polypropylene Conical Tube

Either the Arcturus XT or Veritas Microdissection System

Manual for Arcturus XT or Veritas Microdissection System

Gloves

Lab coat

Eye protection

Lab wipes

ExtracSure Sample Extraction Device (Life Technologies) if using HS LCM Caps

Cap Tool

**LCM instruments used for development and testing of protocol**

The Arcturus XT model was built on the Nikon Ti base with software version 3.1.0.0 and fluorescence module X-Cite Series 120 PC.

The Veritas was model number 704 which included both the UV cutting laser and fluorescence.

**Safety:**

Perform all bench work until slides are loaded on the instrument wearing gloves, lab coat, and proper eye protection. In the Microdissection Core Facility at the National Cancer Institute (NCI) we do not handle any part of the LCM instrument except for the stage with gloves. Resume wearing personal protective equipment (PPE) when handling the cap and for the extraction of RNA after microdissection has been completed. This segregation is important for user safety and limits the potential for contamination of the samples. Change gloves frequently. Finally, dispose of all waste material and chemicals in the proper way according to local regulations.

**Setup of Work Area**

**Cold Storage of Slides and Extracted RNA:**

A properly sized, covered insulated box half full of with dry ice is needed to store the slides that will be microdissected during the day as well as the tubes containing the extracted RNA. Only remove the slides for that day’s use from the -80° C. Do not put the slides directly on the dry ice. Protect the slides from condensation by using either smaller slide boxes or Pap Tubes to store them. When removing the slide to start dissection, reseal the slide containers quickly.

**Cleaning:**

In order to ensure a RNase free environment clean your work area with 70% ethanol, followed by RNase Away, and then with 70% ethanol to remove residual RNase Away. When preparing the 70% ethanol use RNase free water. The work area includes the bench, the hood, and any other surface a cap or tube may touch including the inside of the incubator. For delicate equipment, such as the stage of the microscope or the pipettes used during the procedure, use pre-wetted lab wipes. Do not use a spray bottle to avoid getting either the ethanol or the RNase Away reagent into delicate parts of the equipment.

Use only RNase free disposables. Ensure that the RNase removal agent does not directly touch the tissue or the tubes because it may interfere with the subsequent processing of the samples.

Clean the glass support slides, because contaminating dust has often been found on slides obtained from the manufacturers labeled “Precleaned“. Handle slide only at the frosted end. One support slide is needed for each framed membrane slide. Store clean slides in a sealed slide box until use. Follow the following steps:

1. Wipe the slide with lens paper.
2. Clean the slide with 70% ethanol and lab wiper
3. Clean the slide with RNase Away. Let sit. Use second wipe to remove excess solution
4. Clean slide with 70% ethanol
5. Allow all the ethanol to evaporate before use.

**Staining**

Before staining make sure the LCM instrument is ready to use.

In the hood setup clean 50 ml conical or pap tubes for staining /dehydration.

In this study because of the GFP tag only a quick dehydration was needed to see the cells of interest.

Five tubes were set up:

1. 70% ethanol (use RNase-free water to dilute 100% EtOH)
2. 95% ethanol
3. 100% ethanol
4. 100% ethanol
5. Xylene (always handle the xylene in a fume hood)

**LCM procedures and the Cap Road Map Method.**

Final Slide Preparation for Microdissection:

1. Prepare up to three slides; use an appropriate number that can be dissected in a reasonable amount of time. (For this study we used brains that had EGFP to denote the cells of interest, sectioned on PEN Frame Metal Slides. See paper and Supplemental protocol 1). The dehydration procedure optimized for these conditions is as listed below. You can use any appropriate staining procedure for this step.
2. Have your support slides clean and dry before preforming the Dehydration procedure.
3. Keep Slides on Dry Ice in a closed container to limit condensation and frost formation on mounted tissue until use.
4. Using the solutions prepared on the day of use, in a fume hood perform the following steps:
   1. 75% Ethanol for 30 sec
   2. 95% Ethanol for 5 sec
   3. 100% Ethanol for 5 sec
   4. 100% Ethanol for 5 sec
   5. Xylene for 30 sec
   6. Allow Xylene to completely evaporate
      1. This usually takes 2-5 min.
      2. Angle slides on clean surface tissue side out.
5. When dry place frame membrane slide tissue side down on the support slide and load on to instrument (See Fig. 2).

Setup For Arcturus XT Microdissection System

1. Clean the microscope stage (if necessary).
   1. When the instrument is off, the stage is in the “unload” position.
   2. The stage can be better accessed by tilting the light tower:
      1. Loosen the thumbscrew on light tower at the back of the instrument.
      2. Gently tilt the light tower back to its stop point.
      3. Clean the stage as described in the cleaning section (above).
      4. Gently tilt light tower to original upright position and retighten thumbscrew.
2. Start the instrument by turning the components on in the following order:
   1. Turn on the Nikon Base, the switch is located on in the lower right of the microscope. Allow it to initialize. This is indicated when the Status Display Panel (LED) on the front of the microscope shows the z-position.
   2. Turn on the florescence source.
   3. Turn on the computer and log into Windows.
   4. Turn on the laser control unit. (The switch is on the left side of the box labeled Arcturus XT to the left of the Nikon base.)
3. Setting up the light path. Ensure that all of the manual settings in the light path of the microscope are in the proper position: These are listed from top to bottom of the scope. When the XT software is opened it assumes all of these are set to the bright field positions (see below).
   1. Illumination source sliders (ensure either all the way in or out).
      1. Diffuser, IN, Labeled “D”, left back.
      2. Neutral Density, Out, Labeled “ND”, left front.
      3. Green Interference Filter, OUT, Labeled “GIF” right rear.
      4. Neutral Color Balance, IN, Labeled “NCB”, right front.
   2. DIC polarizer slider, Labeled “T-P2 filter” is out of the light path (if scope has optional DIC feature).
   3. Condenser wheel is in position “A” for brightfield if (if scope has optional DIC and/or Phase contrast module).
   4. “Fluorescence filter turret” is in an open position (not labeled with a filter indicator). The florescent filter turret is located under the objectives.
   5. Manual fluorescent shutter is in the “O” Open position. The lever is located next to the filter wheel on the right side of the scope.
   6. DIC Analyzer slider , Labeled “T-A2”, is in the out position. It will be the first stop point as the slider is pulled out. This is located below the fluorescence filter turret.
   7. The “Magnification Selection Knob” is on the “Front Operation Panel” of the scope. Rotate it to 1x position, pointing up.
4. Start the Arcturus XT software by selecting the Arcturus XT icon.
5. Type in your user name and password.
6. Set microscope parameters (the parameters shown below were used in this study):
   1. In the “Inspect ” tool panel click on the “i” to open the “Inspect Options” tool box.
   2. In the “Illumination” tab ensure the following:
      1. For the Microscope Lamp area.
         1. Select the “On” Check box.
         2. The “Intensity” slider set to 46.
         3. The “Camera Gain” slider set to 320.
      2. The “White Balance” Auto check box should be selected.
      3. In Diffuser Setting select the “IN” radio button.
      4. Select “OK” to save changes.
7. Loading Slides and Caps: Image needed.
   1. In the “Setup” tool panel click “Present Stage” button.
   2. The stage will move to the load position.
   3. Click on button “i” in the “setup panel”. This opens the “Load Options” box.
   4. In the “Load Options” toolbox with the “Slides” tab selected.
      1. Click the check boxes that correspond to the slide slots you are using.
      2. Select the radio button that corresponds to “Framed” for each loaded slide.
      3. If desired add “SlideName” to Attributes Area.
   5. Select “Caps” tab in the “Load Options” box.
      1. Choose “Macro” caps radio button regardless of type of the caps loaded.
      2. Select Load Cap check boxes corresponding to caps in cassette to be loaded.
   6. With Gloves, Load the following:
      1. PEN Membrane Frame Slides with glass support slides.
         1. Place the slides in the 3 parallel slots, with the label to the right. The slides should under the tabs on the front of each slot resting on the spring locking mechanism.
         2. Use the tension button to drop the slides into place. They should be securely in the slot an parallel to the stage.
      2. Load a cassette of CapSure Caps into the Cap Cassette area perpendicular to the Slide Slots. Tilt the cassette in the direction of the arrow on the stage and slide them into the slot. They will be flat when loaded.
   7. Remove Gloves.
   8. Click “OK” at the bottom of the “Load Options” toolbox to move the stage to the center of the first loaded slide.
8. Acquiring overview images (Important for finding Regions of Interest).
   1. To ensure that the slide you want to image is selected.
      1. The active slide button is dark in the “Slide” area.
      2. To change the active slide click on the button (“A”,”B”,”C”) corresponding to the slide slot label on the instrument.
   2. Click on the approximate location of your tissue in the slide overview image area. Without an overview image this area is a large gray box in the middle of the bottom of the XT software window.
   3. In the “Inspect” tool panel:
      1. Click on 2x to change objective. If not already set at 2x
      2. Adjust exposure time using the "Brightness” with the up and down arrow buttons.
      3. Adjust the “Focus” using the arrows or the “eye” button for auto focus.
   4. When satisfied with the “Main Image” right click in the slide overview image to bring up “right click menu”:
      1. Select “Remember Settings”.
      2. The “Overview” pop up box will ask for conformation. Click the “Yes” button.
      3. The right click menu will reappear select “Reacquire Overview Image”.
   5. The instrument will take and “stitch” (combine) 45 images. When this processes is completed the image will appear in the slide overview image area.
   6. Repeat for each slide Loaded on the instrument.
9. Locate the Regions of Interest, in this case the Supraoptic Nucleus (SON), and save location.
   1. Using the Slide Overview Image click on the approximate location of your target cells. The stage will move to center the main image of the tissue on that location.
   2. Find the precise location of your cells of interest on the main image screen:
      1. Focus the image.
      2. Center your cells on the stage using either the trackball, keyboard arrow keys or when “Move Stage” enabled, the mouse can be used.
      3. Change objective to the “Map Objective”, 20X in this case This is determined by the size of the region of interest How to choose your map.
      4. Focus the image.
   3. Store the position by clicking the “+” button in the “Stored Position” controls at the bottom the “Select” Tools panel.
      1. If it is the first stored position a “1” will appear between the “<” and the “>” buttons.
      2. When subsequent positions are stored the number between the “<” and “>” will increase.
   4. Repeat this until all regions of interest have been stored.
10. Locate UV laser and test on the map objective.
    1. Move to tissue that is not to be collected and focus.
    2. Click on the “Laser Bypass” button.
    3. Click on the “i” in the Microdissect tool panel to open the “Microdissect Options” toolbox.
    4. Click on the “UV Locate” Tab.
    5. Follow directions for “Locating UV laser” in “UV Locate” tab.
    6. After the UV laser has been located click “OK” at the bottom of the “Microdissect options” box.
    7. These steps only need to be preformed for the first dissection.
11. Place microdissection cap in mapped location using the LCM Cap Road Map (for 20x magnification with a macro cap we use the map in Figure 4A) and set up the capture laser:
    1. Make sure “Map objective” of 20x is selected.
    2. In the “stored positions” controls area click on “|<” button to move the stage back to the first stored position.
    3. Positioning the Stage to Capture a saved region using the LCM Cap Road Map.
       1. Choose where on the cap map the first region is to be collected.
       2. Activate the Main image by clicking on it.
       3. Use Control + Arrow as indicated by the cap map, illustrated in Figure S1A, to move the main image to the center position on the cap. It may be convenient to print out the cap map and cross off regions, as they are collected.
    4. Place the Cap at region Center.
       1. Choose one of the following:
          1. In the “Microdissect Tool Panel” click the “Place cap on live image location” button
       2. The cap robot arm will now place the active cap. This is either the cap on any slide or the next cap in the cap cassette. Directly after the move the instrument will automatically locate the laser for the current objective.
    5. Return to stored position, area for collection, by clicking on the number between the “<” and “>” in the stored positions controls area.
    6. Focus “Main image”.
    7. If this is the first capture on the cap, set up the capture laser for capturing area. (This is for framed membrane slides, for other types of slides or if there problems see notes on setting up spots)
       1. Click on the “i” in the Microdissect tool panel to open the Microdissect Options tool box.
       2. At this time you should have only one Options tool box open.
       3. Click on the IR Spot Sizes Tab.
       4. Ensure that the spot size selected in the IR spotsize control box in the Select tool panel is the same as the IR Spot sizes tap and that any changes that are made are in the selected column.
       5. Uncheck the Auto move stage checkbox.
       6. Choose a spot diameter that is smaller than the item you are trying to collect.
       7. Select power and duration good starting point is usually:
          1. Power: 30 mW.
          2. Duration: 20 tenths of a millisecond.
       8. If available, in an area with no tissue click the “Test IR Shot” button to inspect the capture spot.
       9. Click “OK” on the bottom of the Microdissect options box to save changes.
    8. Florescence
       1. Rotate fluorescence filter turret to the proper filter cube
       2. Open the electronic shutter florescence by clicking “Turn Florescence On” in the inspect tool panel.
12. Select Cells
    1. Click on the “Draw freehand” tool in the Select tool panel.
    2. Use the mouse or pen tablet screen to outline the cells of interest.
       1. Depress the right button to start line release it to stop drawing.
       2. For individual cells cut a little outside of the cell in order to not damage it with the UV laser .
       3. Stop the line slightly before completing the outline to allow for the ends to snap too each other.
       4. Make sure there is a IR spot in each collected area.
13. Microdissect Selected Cells
    1. In “Microdissect” tool panel Click on “IR Capture and UV Cut button”.
    2. Allow instrument to finish preforming the microdissection before continuing.
14. Repeat placing the cap, selecting the cells of interest and microdissecting until the cap is full or all regions are collected.
15. Offloading The Cap:
    1. When finished working with the cap, click “Move cap to QC station” button to move the cap to the QC station.
    2. The cap will be in the QC station corresponding to its position in the cap cassette.
    3. Inspection of the cap is possible in with the 2x-20x objectives.
    4. To remove the cap from the stage for digestion either click “present stage” in the Setup Tool Panel or if finished using the instrument click File 🡪 Exit. The stage will move to the load position and is accessible by the cap-handling tool.

**Preparation of LCM isolated RNA**

The PicoPure extraction buffer can be aliquoted to 2.0 ml screw top Sarstedt tubes (Sarstedt Inc., NC, USA) for easier handling.

Preheat the digestion oven to 42° C.

If using CapSure HS tubes preheat the incubation metal blocks

**Notes for the XT software:**

1. When using “options” boxes in the Arcturus XT software you must click the “OK” for the settings to take effect. Clicking the x in the corner will not record the changes when the box is closed.
2. Only have one toolbox open at a time. When the XT.
3. In each tool panel there is an button labeled “i” that will open the respective “options” box.

**Using the Cap map method with The Veritas instrument:**

The Cap Map can be applied to the Veritas Microdissection system as well as the Arcturus XT. Some differences between the instrument that need to be considered are:

1. Movement of the stage: on the Arcturus XT, shift 🡪, moves the stage a whole field of view whereas on the Veritas, shift 🡪, moves the stage by only half a field of view so to use the map you must double the number times selecting the arrow key to move to the desired position.
2. On the Veritas the IR capture laser must be focused before capturing cells.
3. The Veritas will detect the slides and caps loaded in the instrument and also take overview images automatically.
