# Supplementary material for: Analysis of Transcription Factor mRNAs in Identified Oxytocin and Vasopressin Magnocellular Neurons Isolated by Laser Capture Microdissection
Source: PLoS One. 2013 Jul 24;8(7):e69407. doi: 10.1371/journal.pone.0069407 (PMC3722287; doi:10.1371/journal.pone.0069407)
Supplement: Table S3 — Primers used for qRT-PCR of Oxt and AvP MCN mRNA. (DOC) [file pone.0069407.s004.doc]

Table S3. Primers used for qRT-PCR of Oxt and AvP MCN mRNA

| *Gene Name* | *Primers* | *Primer Sequence* | *Amplicon Length* | *Tm (°C)* |
| --- | --- | --- | --- | --- |
| Oxt | Sense | GGCATCTGCTGTAGCCCG | 62 bp | 84.15 |
|  | Antisense | AAGGCAGACTCAGGGTCG |  |  |
| Avp | Sense | TGCCTGCTACTTCCAGAACTGC | 77 bp | 80.80 |
|  | Antisense | AGGGGAGACACTGTCTCAGCTC |  |  |
| RORA | Sense | TGCCACCTACTCCTGTCCTC | 112 bp | 83.05 |
|  | Antisense | ATCTCGAGACATCCCCACAG |  |  |
| CREB3 | Sense | TAGGATGCCCAGGAACTCAC | 136 bp | 80.02 |
|  | Antisense | CTTCCCATTCCTTCTGTGGA |  |  |
| Clock | Sense | AGAACTTGGCGTTGAGGAGT | 115 bp | 79.33 |
|  | Antisense | ATCGAACCTTTCCAGTGCTT |  |  |
| c-Jun | Sense | TTGACCAGAAGATGGTGCAG | 124 bp | 80.72 |
|  | Antisense | CACAGCGCATGCTACTTGAT |  |  |
| GAPDH | Sense | CAGAGCTGAACGGGAAGCT | 125 bp | 81.30 |
|  | Antisense | CTTCACCACCTTCTTGATGTCA |  |  |
