# Supplementary material for: Analysis of Transcription Factor mRNAs in Identified Oxytocin and Vasopressin Magnocellular Neurons Isolated by Laser Capture Microdissection
Source: PLoS One. 2013 Jul 24;8(7):e69407. doi: 10.1371/journal.pone.0069407 (PMC3722287; doi:10.1371/journal.pone.0069407)
Supplement: Table S4 — Raw Ct Values for qRT-PCR of Oxt and Avp MCN mRNA. (DOC) [file pone.0069407.s005.doc]

Table S4 Raw Ct Values for qRT-PCR of Oxt and Avp MCN mRNA

|  | *Oxt MCN Samples* | | | | | | *Avp MCN Samples* | | | |
| --- | --- | --- | --- | --- | --- | --- | --- | --- | --- | --- |
| *Gene Name* | *Sample 1* | | *Sample 2* | *Sample 3* | *Sample 4* | *Average* | *Sample 1^†^* | *Sample 2* | *Sample 3* | *Average* |
| Oxt | 21.64 | | 23.90 | 23.34 | 23.93 | 23.20 | 25.61 | 27.73 | 27.95 | 27.10 |
| Avp | 24.66 | | * | 28.70 | 27.82 | 27.06 | 21.39 | 22.40 | 23.93 | 22.57 |
| RORA | 28.46 | | 30.93 | 33.90 | 34.16 | 31.86 | 32.59 | 32.64 | 33.48 | 32.90 |
| CREB3 | 35.29 | | * | 34.56 | 35.55 | 35.13 | 36.33 | 32.93 | 33.88 | 34.38 |
| Clock | 32.44 | | 32.91 | 34.67 | 35.91 | 33.98 | 33.87 | 33.62 | 33.80 | 33.76 |
| c-jun | 33.59 | | 31.94 | 35.54 | 33.92 | 33.75 | 34.86 | 33.47 | 34.46 | 34.26 |
| GAPDH | | 25.61 | 25.94 | 29.27 | 29.24 | 27.52 | 25.35 | 26.94 | 28.33 | 26.87 |
| No RT | 36.60 | | 36.00 | 36.94 | 31.85 | 35.35 | * | 28.58 | 32.31 | 30.45 |

* mRNA was undetectable

^†^ sample 1 is pooled RNA from two 2.0VPI-EGFP rats
