# Supplementary material for: Analysis of Transcription Factor mRNAs in Identified Oxytocin and Vasopressin Magnocellular Neurons Isolated by Laser Capture Microdissection
Source: PLoS One. 2013 Jul 24;8(7):e69407. doi: 10.1371/journal.pone.0069407 (PMC3722287; doi:10.1371/journal.pone.0069407)
Supplement: Table S5 — Number of Cells Collected by LCM and Amount of RNA Extracted from Each Rat. (DOC) [file pone.0069407.s006.doc]

Table S5. Number of Cells Collected by LCM and Amount of RNA Extracted from Each Rat

| MCN Sample # | Number of Cells Collected per Rat | Amount of RNA Extracted (ng) |
| --- | --- | --- |
| Oxt MCNs | | |
| 1 | 1158 | 82.5 |
| 2 | 448 | 121.5 |
| 3 | 637 | 119.3 |
| 4 | 686 | 89.8 |
| 5 | 2043 | 420 |
| 6 | 1120 | 157.5 |
| 7 | 1195 | 186 |
| 8 | 1444 | 165 |
| 9 | 1221 | 114 |
| Average | 1105.8 | 161.7 |
| Avp MCNs | | |
| 1* | 1455 | 103.0 |
| 2 | 489 | 79.7 |
| 3 | 1082 | 89.6 |
| 4 | 1099 | 99 |
| 5 | 1557 | 102 |
| 6 | 1695 | 222 |
| 7 | 1103 | 81 |
| Average | 1211.4 | 110.9 |
